# Precision of Medication Therapy Problem Identification and Classification amongst Primary Care Clinic Pharmacists

**DOI:** 10.3390/pharmacy9040179

**Published:** 2021-11-03

**Authors:** Nicholas Cox, Bryce Ashby, Bradly Winter, Gregory Stoddard, Joanne LaFleur, G. Benjamin Berrett, Kyle Turner

**Affiliations:** 1 College of Pharmacy, University of Utah, Salt Lake City, UT 84112, USA; joanne.lafleur@pharm.utah.edu (J.L.); kyle.turner@pharm.utah.edu (K.T.); 2 Pharmacy, PeaceHealth Southwest Medical Center, Vancouver, WA 98664, USA; bashby@peacehealth.org; 3 Pharmacy Services, Intermountain Healthcare, Taylorsville, UT 84123, USA; bradly.winter@imail.org; 4 School of Medicine, University of Utah, Salt Lake City, UT 84112, USA; greg.stoddard@hsc.utah.edu; 5 Pharmacy Services, University of Utah Health, Salt Lake City, UT 84112, USA; golden.berrett@hsc.utah.edu

**Keywords:** medication therapy management, comprehensive medication management, clinical pharmacy, implementation science, pharmacist

## Abstract

This study assesses the level of agreement on medication therapy problem (MTP) identification and classification between primary care, ambulatory care pharmacists within a health-system that recently implemented system-wide pharmacist provision of comprehensive medication management (CMM) services. Twenty standardized case vignettes were created and distributed to pharmacists who reviewed each case and identified and categorized MTPs. Outcomes include the number of MTPs identified, identification (yes/no) of specific MTPs within each case (e.g., need for a statin), and Pharmacy Quality Alliance (PQA) category used when classifying MTPs. The level of agreement on MTP identification/categorization was measured using intraclass correlation coefficient (ICC) and interpreted using the Landis and Koch interpretation scale. “Moderate agreement” was observed for the number of MTPs identified by pharmacists (ICC equal to 0.45; 95% confidence interval [CI]: 0.31 to 0.65). In approximately one-half of opportunities, the pharmacists agreed perfectly on the number of MTPs; in approximately one-third of opportunities, the number of MTPs identified varied by 1; and approximately one-tenth of the time, the number of MTPs varied by 2. In regard to the MTP identification (yes/no) and categorization, percent agreement was ≥73% across all MTPs. The results support the need for further training and education and provide the information necessary to target specific disease states.

## 1. Introduction

Appropriate, effective, and safe medication use can decrease patient burden and improve overall health. A 2007 Institute of Medicine report identified medications as the most widely used therapeutic intervention with the ability to provide immense benefit, or harm [1]. Medications are widely used to treat and prevent a myriad of diseases. More than 3.5 billion prescriptions are written every year in the US [2], with an estimated yearly cost in 2018 of $528.4 billion for drug-related morbidity and mortality [3]. Preventable medication-related adverse events among ambulatory care comprise up to 45% of reported harmful incidents [4]. To maximize benefit, minimize harm, and decrease healthcare spending, it is crucial to evaluate the utility of each medication on a patient’s medication list.

Comprehensive medication management (CMM) is a process developed to offer a framework for identifying and categorizing medication therapy problems (MTPs) [5]. Similar formal processes have been discussed and practiced since at least 2003; one example is medication therapy management (MTM), which served as a predecessor for CMM [6]. While formally distinct, MTM and CMM are closely related with much overlap in frameworks, especially as CMM development has origins from the MTM framework. The terms are often used interchangeably in literature to describe a holistic pharmacist patient care process. In an attempt to standardize and advance the pharmacists’ patient care process, CMM provides a “…standard of care that ensures each patient’s medications (whether they are prescription, nonprescription, alternative, traditional, vitamins, or nutritional supplements) are individually assessed to determine that each medication is appropriate for the patient, effective for the medical condition, safe given the comorbidities and other medications being taken, and able to be taken by the patient as intended” [5]. The Pharmacy Quality Alliance (PQA) is a national quality organization that provides initiatives to support improved medication use. As part of CMM, the PQA has published a framework for the classification of MTPs that emphasizes four overarching categories: Indication, Effectiveness, Safety, and Adherence [7,8]. These methods and frameworks have been adopted and are supported by numerous professional organizations including the American College of Clinical Pharmacy (ACCP) [9]. Versions of the same PQA framework were also utilized in MTM studies.

The implementation of a standardized pharmacist patient care process has shown beneficial clinical, economic, and humanistic outcomes [6,10]. Ramalho de Oliveira et al. demonstrated that 55% of patients enrolled in a system that practiced MTM met their therapeutic goals and that the estimated return on investment was $1.29 per $1 spent on services [6]. Additionally, patients were highly satisfied with the services and greater than 95% agreed that their overall well-being was enhanced. Isetts et al. prospectively compared patients that received MTM services and patients that did not and found 71% of MTM patients met hypertension therapeutic goals compared to 59% of the comparison group and 52% of MTM patients versus 30% of comparators met their cholesterol management goals [10]. Additionally, decreases in total health expenditures were noted from $11,965 per person to $8197. This reduction in expenditures exceeded the cost of the services provided. While healthcare costs have likely changed since these 2008 and 2010 publications, they continue to illustrate that quality pharmacist patient care processes have been shown to influence patient, as well as economic outcomes in a positive manner. However, a 2015 meta-analysis of MTM interventions identified that the evidence of benefit is insufficient, in part, due to inconsistency of practices [11]. To the authors’ knowledge, no published systematic reviews have similarly assessed the benefit of formal CMM.

Precision and consistent practice among pharmacists providing CMM services are critical to delivering and evaluating a quality CMM program. In mid-2017, the University of Utah Health Pharmacy Primary Care Services (PPCS) began to implement CMM in response to the professional call for adoption of standardized practice and as a solution to inconsistent practices and outcomes that were observed among pharmacy practice sites. This undertaking has included substantial clinical pharmacy education and training in the form of group instruction, coaching, and practice management changes [12]. To ensure high fidelity to the CMM model and increase the likelihood of positive clinical, economic, and humanistic outcomes observed at other institutions, quality assessment of this clinical pharmacy CMM program is essential [13,14,15]. Pharmacists’ ability to consistently identify MTPs is a critical first step and skill that will dictate subsequent actions that will improve outcomes. Additionally, given the data-driven reliance of past studies and potentially the decisions of stakeholders on the types of MTPs being identified by pharmacists, the pharmacists’ ability to consistently categorize MTPs is an essential skill that will impact the ability to evaluate and improve MTP identification and intervention.

In this setting, fidelity to best practice can be challenging to assess. Accuracy, being a measure of the degree of proximity to a correct answer, is difficult to assess because of the relative subjectivity of a correct answer in clinical scenarios. Precision, being the degree of consistency independent of a correct answer becomes a more reasonable measure. Therefore, a primary step in quality assessment is to evaluate the consistency of MTP identification and classification [16]. Inter-pharmacist consistency in the identification and documentation of MTPs is a measurable outcome that can be used to assess precision. A high level of agreement ensures uniformity and reliability with the goal of producing the quality outcomes that the CMM program was implemented to achieve. While other studies have assessed precision among pharmacists, to our knowledge, no study has assessed the precision of pharmacists providing CMM utilizing the Pharmacy Quality Alliance (PQA) framework. The purpose of our work is to assess the level of agreement between primary care, ambulatory care pharmacists in the University of Utah Health system in the identification and categorization of medication therapy problems by piloting a novel case-based analysis technique.

## 2. Materials and Methods

### 2.1. Setting and Participants

University of Utah Health PPCS services 14 primary care clinics throughout the state of Utah with 16 primary care clinical pharmacists, three post-graduate year 1 (PGY1) ambulatory-focused residents, one post-graduate year 2 (PGY2) ambulatory care resident, nine pharmacy technicians, and one pharmacy intern. In 2017, the PPCS team implemented CMM throughout all 14 clinics. During implementation, significant education and training was provided to all team members in the form of group instruction, coaching, and practice management changes [12]. Residents had all completed PPCS ambulatory care rotations prior to the study and received training in CMM and MTP categorization.

### 2.2. Evaluation Strategy and Data Collection

The investigators designed 20 patient case vignettes that were distributed to each primary care pharmacist, including the 4 pharmacy residents. Participants were instructed to work through each case and document all MTPs they identified and to categorize them according to the PQA framework [8]. There was no limit placed on the number of MTPs that could be identified for each case. Baseline characteristics were collected for each participating pharmacist. All classifications and data were captured using Qualtrics software [17]. Pharmacists completed the work individually and were instructed to avoid discussing cases until the data collection was completed. Online resources were available to the pharmacists as they would be in actual practice. All participants were given a 15-min orientation to the study and then were allotted 2.5 h to complete their review of all cases. The time limit was placed to encourage efficiency as is required in actual practice, but participants could request additional time as needed.

### 2.3. Outcomes

The primary outcomes are the level of agreement (percentage agreement and interrater agreement) on: (1) the number of MTPs identified within each case, and (2) the identification (yes/no) of specific MTPs within each case. For example, in case #1, a patient has diabetes but is not being treated with a statin medication. The primary outcome will evaluate the proportion of participants who identified this as a problem within case #1. Secondary outcomes include the mean number of MTPs identified per case, level of agreement of MTP identification by disease state (e.g., diabetes), and the level of agreement on the PQA categorization of identified MTPs by disease state.

### 2.4. Case Vignettes

Cases were designed using information from patient electronic medical records with the intent to emulate realistic patient scenarios and contain the information necessary to perform a CMM review as is done in practice. Patient identifiers were omitted from all cases. Case format was standardized to each contain a medication list, chief complaint, past medical history, pertinent immunization records, laboratory results and vital signs. To minimize investigator bias, the cases were designed to not inappropriately bias the reviewers towards target MTPs by including a realistic blend of comorbidities, social history, medications, and immunization history. In order to ensure that case vignettes were representative of an actual population, the cases were designed in a manner that maintains a similar distribution of disease states observed by University of Utah PPCS pharmacists and a similar distribution of MTP categories that has been observed in published reports (Table 1) [6,18,19]. The reports used, although from 2003 to 2010, were chosen due to their detailed reporting of MTPs by problem using the PQA framework. For MTPs without published data, we assumed the prevalence to be no more than the lowest of the published MTPs. Published data indicates that the percentage of patients with at least one MTP in a given population is 76.7% to 90.1% and with a mean of 1.1 to 2.4 MTPs per patient [10,18,19]. Our 20 patient cases were created in a manner representative of these data with 95% of our cases containing at least one MTP and an average of 1.75 planned MTPs per patient. Prior to case distribution, all cases were reviewed by an independent pharmacist with expertise in CMM who was not a participant in the study. This expert reviewed each case for clinical relevance or missing information, and to ensure each case used language reflective of health records which did not inappropriately “lead” participants to identify certain MTPs nor categorize MTPs in a unified fashion. Cases were amended based on the input from the independent pharmacist until an agreement was reached for each case.

### 2.5. Statistical Analysis

Interrater agreement was measured with the intraclass correlation coefficient (ICC), computed with a two-way random effects model, so inferences can be made to pharmacists and clinical cases in general, not only those included in the study. The individual, absolute agreement form of the ICC was used. Interpretation of ICC was conducted using Landis and Koch’s scale (Table 2) [20]. Agreement was based on the “mode” answer choice result for a given outcome. Sample size was calculated based on the primary outcome. Power was limited by the number of ambulatory care pharmacists in the University of Utah Health system, which at this time is 20. Since pharmacists essentially classified each MTP as either present or absent, the quantity of observations per pharmacist was not affected by the prevalence of MTPs in the cases but rather by the number of cases reviewed. As such, the power calculation was a function of the anticipated precision of pharmacist classifications (70%) within the 95% confidence interval (CI). For a 95% confidence interval around an ICC of 70%, we calculated that 18 cases would be needed in order to assess kappa with ±15% precision, using either 19 or 20 pharmacist raters [21].

## 3. Results

### 3.1. Participant Demographics

Of the 20 pharmacists in the health-system, 19 completed the cases and were included in the study. The average years of pharmacy practice was 7.6 with a mode of 2 years of residency training. Of the participants, 63.2% were board certified and 52.7% were female. No participant requested additional time (beyond the allotted 2.5 h) to complete the cases.

### 3.2. Primary Outcomes

Regarding the level of agreement on the number of MTPs identified within each case, the ICC was 0.45 (95% confidence interval [CI], 0.31 to 0.65), which correlates to a “moderate” level of agreement. Overall, 47% of the time, the participants agreed perfectly as to the number of MTPs within each case; 38% of the time, the participants disagreed by ±1; 11% of the time, the participants disagreed by ±2; 2% of the time, participants disagreed by ±3; less than 1% of the time, participants disagreed by ±4; and less than 1% of the time, participants disagreed by ±5.

The overall level of agreement on the identification (yes/no) of specific MTPs within each case was 78% (95% CI, 76% to 79%). For the overall interrater agreement on the identification of specific MTPs within each case, the ICC was 0.30 (95% CI, 0.25 to 0.37), which correlates to a “fair” level of agreement.

### 3.3. Secondary Outcomes

In total, 939 MTPs were identified collectively by the participants. Overall, 140 unique MTPs were identified that included 37 diabetes, 21 hyperlipidemia, 21 hypertension, eight pain, seven osteoporosis, six immunization, five asthma/chronic obstructive pulmonary disease, five mental health, five safety monitoring, five adherence, four smoking cessation, three heart failure, three gastroesophageal reflux disease, two benign prostatic hyperplasia, two obesity, two atrial fibrillation, one neuropathy, one lifestyle change, one constipation and one illicit drug. The overall mean number of MTPs identified per case vignette was 2.1.

**Figure 1:**
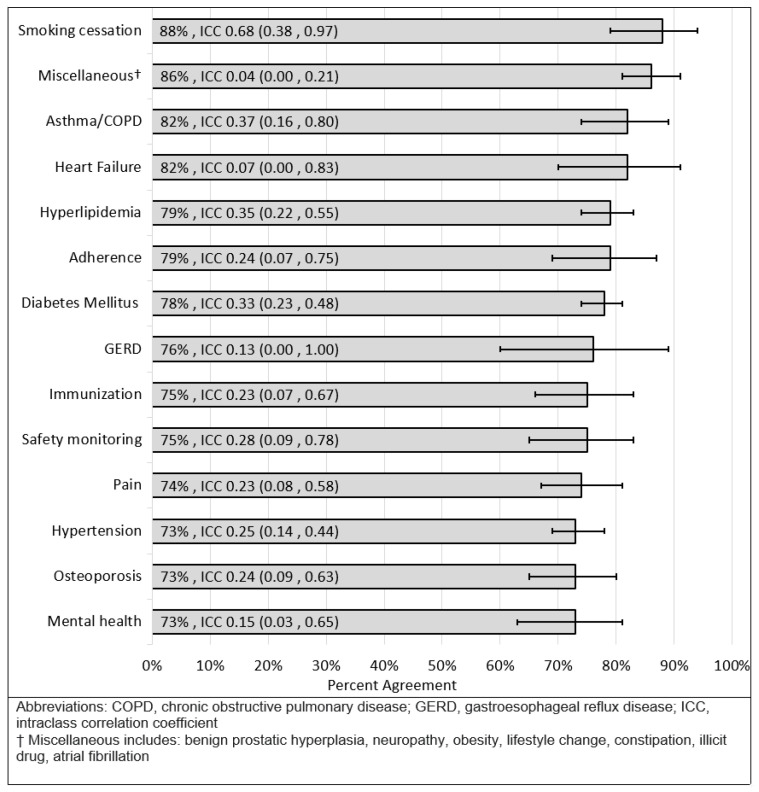
Agreement and 95% confidence interval for MTP identification by disease state.

**Figure 2:**
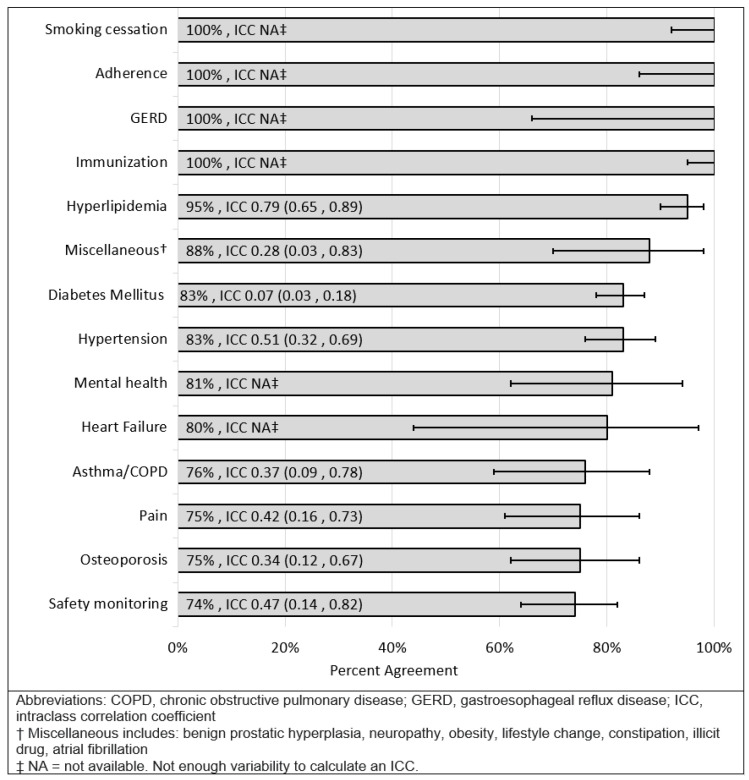
Agreement and 95% confidence interval of MTP Pharmacy Quality Alliance (PQA) categorization by disease state.

Regarding the level of agreement by disease state, percent agreement ranged from 73% (mental health, osteoporosis, hypertension) to 88% (smoking cessation). Figure 1 summarizes the level of agreement in MTP identification by disease state. For participants who identified the same MTP, the level of agreement in the classification of that MTP (according to the PQA framework) varied by disease state. When analyzed by disease state, the percent agreement on classification ranged from 74% (safety monitoring) to 100% (smoking cessation, adherence, gastroesophageal reflux disease, immunization). Figure 2 summarizes the level of agreement in MTP categorization by disease state. The “safety monitoring” grouping in Figure 2 designates when an MTP was identified for an overdue laboratory collection that related to multiple disease states (e.g., basic metabolic panel needed for a patient on metformin, lisinopril, and gabapentin).

Agreement was based on the “mode” answer choice result for a given outcome. 14 individual MTPs identified by participants were excluded from the study due to insufficient information provided to understand the type or nature of the MTP. A small number of participants documented the same problem using 2 different MTPs. In these instances, the MTP was converted to a single MTP for the “identification” analysis but were noted to be not in agreement in the “classification” analysis.

## 4. Discussion

The results of this analysis show that pharmacists demonstrated a moderate level of agreement on the number of MTPs identified within each case; and pharmacists agreed 78% of the time on the identification of specific MTPs within each case. This suggests that, while there is certainly opportunity for improvement, the pharmacists within the University of Utah Health PPCS team do demonstrate a measurable level of agreement and therefore consistency of patient care, at least regarding identification and classification of therapy problems. This is encouraging considering the previously observed inconsistencies in practice [11]. While previously observed inconsistencies likely extend beyond identification and classification, this study represents a first step in evaluating the degree of consistency in aspects of practice.

The level of agreement in MTP identification was lowest amongst disease classes of mental health, osteoporosis, and hypertension. This may be a result of a disease state being less guideline-directed (i.e., mental health) or having conflicting guidelines (i.e., hypertension) or may simply highlight areas of improvement for the institution. Regardless, the results provide objective information for more targeted quality improvement trainings to improve agreement in specific areas of lower agreement. Future institutional training can be focused on these respective disease classes for improved consistency of practice. While this particular result may only be applicable to University of Utah Health, it highlights the potential for these cases and analysis to be a tool that other organizations could utilize.

The level of agreement in MTP categorization was lowest amongst disease classes and categories of safety monitoring, osteoporosis, and pain. This may be a result of the relative subjectiveness of the disease state (i.e., pain), but may also highlight the need for improvement in areas that may be easily overlooked in medication management such as safety monitoring. While there were several disease states that showed 100% agreement on MTP categorization, this may be due to relatively simple classification once a problem is identified (i.e., smoking cessation and immunization). Additionally, since instances of these were relatively low, this may have increased observed agreement. Since agreement was based on the “mode” answer choice result for a given outcome, this study measures agreement not only on those MTPs that were identified, but also those that were not identified. Again, this information can guide future institutional trainings for targeted consistency.

Given this is a pilot study with a novel case-based structure and analysis technique, further validation and testing is required. While the Landis and Koch scale (Table 2) provides some interpretation context to results, the full clinical and operational value will likely require benchmarking via external comparison. Future studies are needed in implementing this tool and analysis in other institutions engaging in CMM practice. Comparisons and analysis will not only provide benchmarking data for quality improvement efforts but can provide demonstration of regional and national consistency in practice; this may aid in leveraging local and potentially national payers for financial reimbursement of CMM services. Future studies are also needed to investigate the association between level of agreement to clinical, humanistic, and financial outcomes.

This study has several limitations. This approach used an unvalidated tool, and as such, it is difficult to determine at this point if it is the cases, the tool, the training, or the approach that contributes to the observed variance. Additionally, given the small sample size, there are inherent limitations in our ability to measure agreement. For example, while ICC is reported for each disease state, given the little variation due to small sample size, the ICC can become less informative. In such circumstances, percent agreement may be more informative than the ICC; therefore, both have been reported. Additionally, residents were not present for the initial 2017 training and implementation and thus may have received training that was not identical to other team members. All residents had, however, completed a rotation with the PPCS team and were well-trained in the practice of CMM. While it is not certain what effect this may have had on the overall results, they were included in the study given they are pharmacist members of the PPCS team. Finally, it is worth noting that this study measures precision and not accuracy. Accuracy is difficult to measure as the “correct” answer is often a subjective decision. This study therefore cannot make any assessment on the accuracy or correctness of the MTP identification and categorization, despite any high level of agreement.

The results of this study indicate that a standardized case-based evaluation of level of agreement in MTP identification and classification can help assess consistency and therefore fidelity to the CMM model. The potential for benchmarking and quality improvement may be useful in providing more consistent and measurable outcomes with lower heterogeneity than what has previously been observed in ambulatory care pharmacy practice. Further validation of this tool amongst external institutions is necessary.

## Figures and Tables

**Table 1:** Planned prevalence of each medication therapy problem (MTP) in the simulated population.

Medication Related Need ^1^	Medication Therapy Problem (MTP) ^1^	Prevalence Observed in Other Populations ^2^	Planned Prevalence (Simulated Sample)	Targeted # of Cases with Given MTP (out of 20 Cases)
Indication	Unnecessary drug therapy	5.7–19.8%	20%	4
	Needs additional drug therapy	15.9–31.4%	30%	6
Effectiveness	Ineffective medication	4.7–15.7%	5%	1
	Dosage too low	13.7–26.1%	25%	5
	Needs additional monitoring	-	5%	1
Safety	Adverse drug reaction	8.3–17.0%	15%	3
	Dosage too high	5.8–6.7%	5%	1
	Needs additional monitoring	-	5%	1
Adherence	Adherence	16.4–31.7%	20%	4
	Cost	-	5%	1

^1^ PQA categories [8]. ^2^ Based on published studies [6,18,19].

**Table 2:** Interpretation of intraclass correlation coefficient (ICC) [20].

Kappa (ICC)	Interpretation
<0	Poor agreement
0–0.20	Slight agreement
0.21–0.40	Fair agreement
0.41–0.60	Moderate agreement
0.61–0.80	Substantial agreement
0.81–1.00	Almost perfect agreement

## Data Availability

Data is contained within the article.
